# Supplementary material for: Inhibition of amyloid beta toxicity in zebrafish with a chaperone-gold nanoparticle dual strategy
Source: Nat Commun. 2019 Aug 22;10:3780. doi: 10.1038/s41467-019-11762-0 (PMC6706415; doi:10.1038/s41467-019-11762-0)
Supplement: Supplementary file 1 — Supporting information [file 41467_2019_11762_MOESM1_ESM.pdf]

# Supplementary Information

## Inhibition of Amyloid Beta Toxicity in Zebrafish with A Chaperone-Gold Nanoparticle Dual Strategy

**Javed *et al.***

## List of Content

**Supplementary Methods**

**Supplementary Tables 1,2**

**Supplementary Figures 1-17**

**Supplementary References**

## Supplementary Methods

### Discrete molecular dynamics (DMD) simulations

DMD is a type of molecular dynamics algorithms where conventional continuous interaction potentials are replaced by optimized step-wise potential functions.[1,2] Comprehensive description of the DMD algorithm was published elsewhere.[49] In brief, the united-atom model was used to represent all molecules where all heavy atoms and polar hydrogen atoms were explicitly modeled. An implicit solvent model was adopted in the current simulations. The interatomic interactions included *van der Waals*, solvation, electrostatic interactions and hydrogen bond. The solvation energy was adopted by the Lazaridis-Karplus implicit solvent model, EEF1.[3] The distance- and angular-dependent hydrogen bond interactions were modeled using a reaction-like algorithm.[4] Screened electrostatic interactions were computed by the Debye-Hückel approximation. A Debye length of 1 nm was used by assuming a water dielectric constant of 80 and a monovalent electrolyte concentration of 0.1 M. The Anderson's thermostat was used to maintain constant temperature.[5]

During the synthesis of AuNP, βCas served as the capping agent. The only one cysteine residue in βCas was not expected to form disulfide bridge under the reducing condition during synthesis. Hence, without chemical modifications βCas adopted its thermodynamically stable conformations on the AuNP surface and the structure of the βCas corona remained independent of the synthesis process of AuNPs. Since our focus was to study the capping of βCas AuNPs and the subsequent ability of βCas corona to bind with Aβ, we therefore modelled only the binding of βCas with a pre-formed AuNP.

We adopted the recently developed Au molecular mechanics force field[6] to model a spherical AuNP with a diameter of 40 Å comprising 1,865 Au atoms. The AuNP force field included both physical and chemical absorption, aromatic and "image" charge interactions. The polarization was modelled by attaching a charged virtual particle (−0.3e) to each metal (0.3e)

atom with a fixed bond length (1.0 Å) as implemented in the GolP force field.[6] Only electrostatic interaction was taken into account for the virtual particle.

The structural coordinates for βCas were obtained from the protein homology prediction server (https://zhanglab.ccmb.med.umich.edu/I-TASSER/). The structural coordinates for Aβ oligomer were obtained from the protein data bank (PDB code: 2NAO), which was a β-sheet rich hexamer structure. Structural coordinates for proteins, basic and acidic amino acids were assigned charges corresponding to their titration states at physiological conditions, i.e. Arg and Lys residues were assigned +1, Asp and Glu were assigned -1, while His was neutral. Counter ions ($Cl^-$) were added to maintain the net charge of the systems zero and accounted for possible counter-ion condensation.[7] All simulations were conducted at 300 K. The periodic boundary condition was applied in all simulations. For each molecular system, 20 independent simulations were performed with different initial inter-molecular distances and orientations to avoid bias. For data analysis, an inter-atomic distance cutoff of 5.0 Å was used to define an atomic contact.

We used a hierarchical clustering program, oc (www.compbio.dundee.ac.uk/downloads/oc), to group similar protein binding poses with the AuNP. Based on an input pair-wise distance matrix that was the center-of-mass distances of the proteins on the AuNP surface in our study, a hierarchical clustering algorithm iteratively joined the two closest clusters into one cluster according to the distances between two clusters. The "cluster distance" was computed based on all pairwise distances between the elements of the two corresponding clusters, which can be the minimum, maximum, or mean of all these values. In this study, we used the mean to compute the distance between two clusters. The centroid structure of each cluster was selected as the one with the smallest average distance to other elements in the cluster.

## Supplementary Tables

**Supplementary Table 1. Zeta potential, hydrodynamic diameter and polydispersity index (PDI) of βCas AuNPs and βCas, before and after incubation with Aβ.**

| **Sample** | **Zeta potential ± SD (mV)** | **Hydrodynamic diameter ± SD (nm)** | **PDI ± SD** |
|---|---|---|---|
| Aβ 0 h | -43.2 ± 2.8 | 3.2 ± 1.6 | 0.261 ± 0.04 |
| Aβ 48 h | -37.4 ± 2.1 | 3565 ± 846 | 0.634 ± 0.13 |
| βCas AuNPs | -11.7 ± 1.8 | 7.5 ± 2.6 | 0.214 ± 0.06 |
| βCas AuNPs+ Aβ | -33.7 ± 2.1 | 39.3 ± 5.4 | 0.345 ± 0.08 |
| βCas | -19.9 ± 2.7 | 156.3 ± 34.4 | 0.324 ± 0.07 |
| βCas + Aβ | -21.7 ± 2.0 | 496.1 ± 114 | 0.674 ± 0.15 |

**Supplementary Table 2. Estimation of protein-AuNP binding energies.** The average potential energies of each molecular system were obtained from the corresponding equilibrated DMD simulations. The AuNP binding affinity difference between βCas and Aβ was estimated based on the obtained average potential energies.

| | **<E> ± SD (kcal•mol$^{-1}$)** |
|---|---|
| Aβ | -79.6 ± 3.5 |
| βCas | -595.5 ± 7.0 |
| Aβ+AuNP | -97.3± 4.0 |
| βCas+AuNP | -807.1 ± 11.1 |

$$\Delta\Delta G \sim (\langle E_{\beta Cas\text{-}AuNP}\rangle - \langle E_{A\beta\text{-}AuNP}\rangle) - (\langle E_{\beta Cas}\rangle - \langle E_{A\beta}\rangle)\text{: } -193.9\ (\text{kcal}\bullet\text{mol}^{-1})$$

## Supplementary Figures

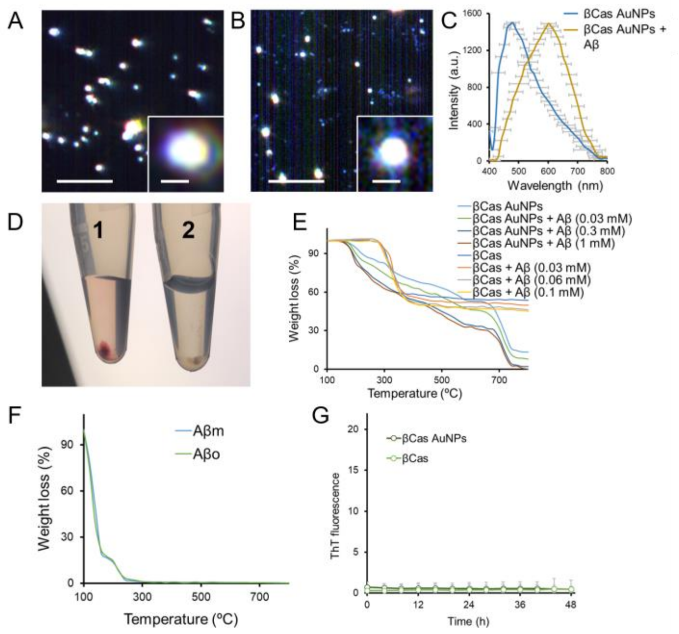


**Supplementary Figure 1. *In vitro* inhibitory interaction of βCas AuNPs with Aβ.** Hyperspectral imaging (HSI) of βCas AuNPs (A) before and (B) after binding with Aβ while (C) presents the SPR spectra and red shift (~100 nm) of βCas AuNPs after binding with Aβ (n=3). (D) Images of βCas AuNPs (1) and βCas (2) pellets after binding with Aβ. (E) TGA curves of βCas AuNPs or βCas alone and after binding with different concentrations of Aβm or Aβo. (F) The TGA curves of Aβm and Aβo were similar in shape. (G) ThT assay for βCas AuNPs and βCas alone as controls (n=3). Scale bars in A and B are 10 µm while 0.5 µm in the insets. Error bars represent the standard deviation. Source data are provided as a Source Data file.

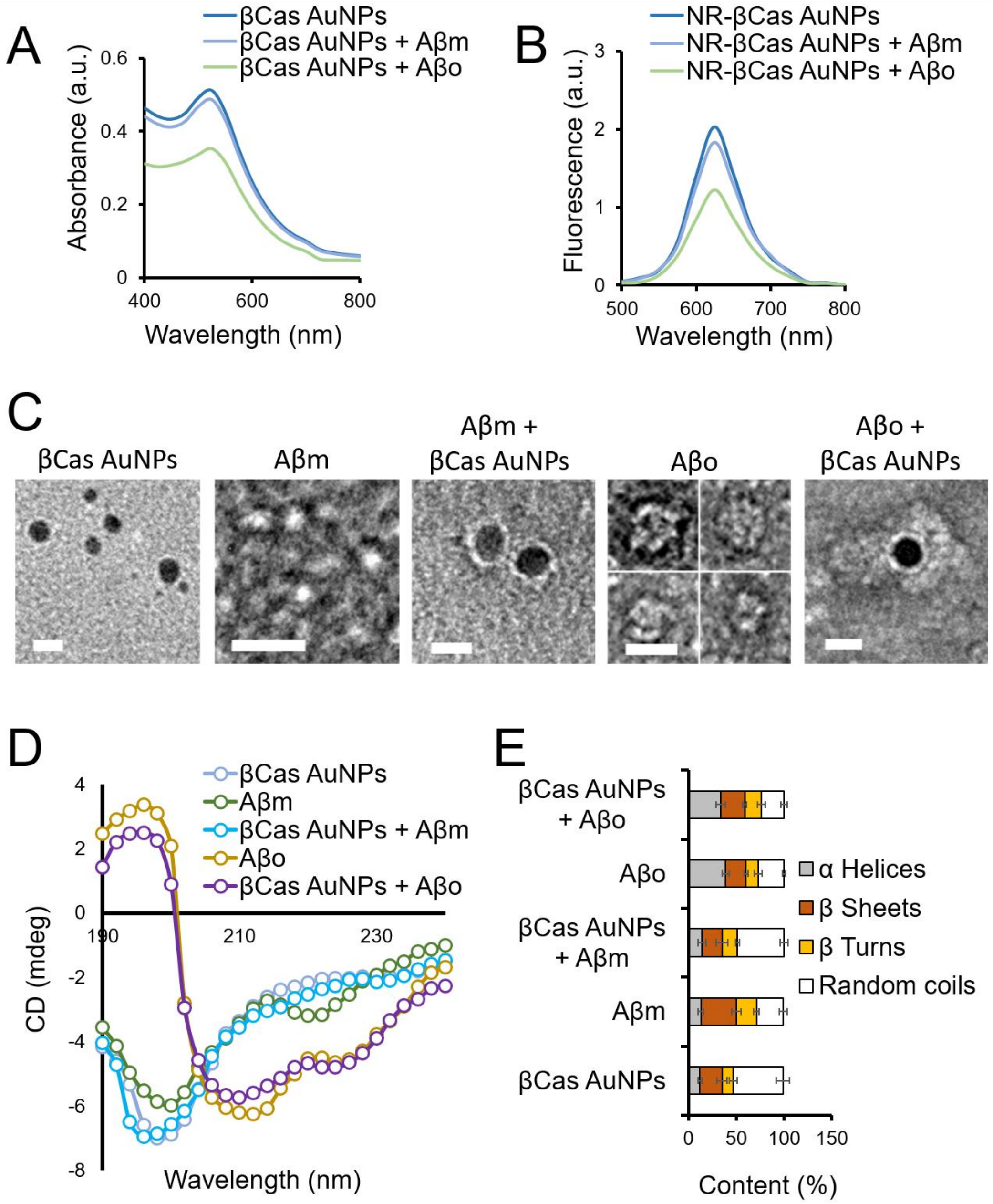


**Supplementary Figure 2. Differential binding of βCas AuNPs with Aβm vs Aβo.** (A) The UV-SPR spectrum of βCas AuNPs was supressed significantly after binding with Aβo as compared to Aβm. (B) Similarly, the fluorescence spectrum of NR-βCas AuNPs was significantly supressed after binding with Aβo. (C) TEM shows corona formation on βCas AuNPs after incubation with Aβo but not with Aβm. (D) CD spectra and percentage secondary structure (E) of βCas AuNPs before and after binding with Aβm and Aβo (n=3). βCas AuNPs displayed a greater affinity for the Aβo corona. Scale bars in TEM the images are 5 nm. Error bars represent the standard deviation. Source data are provided as a Source Data file.

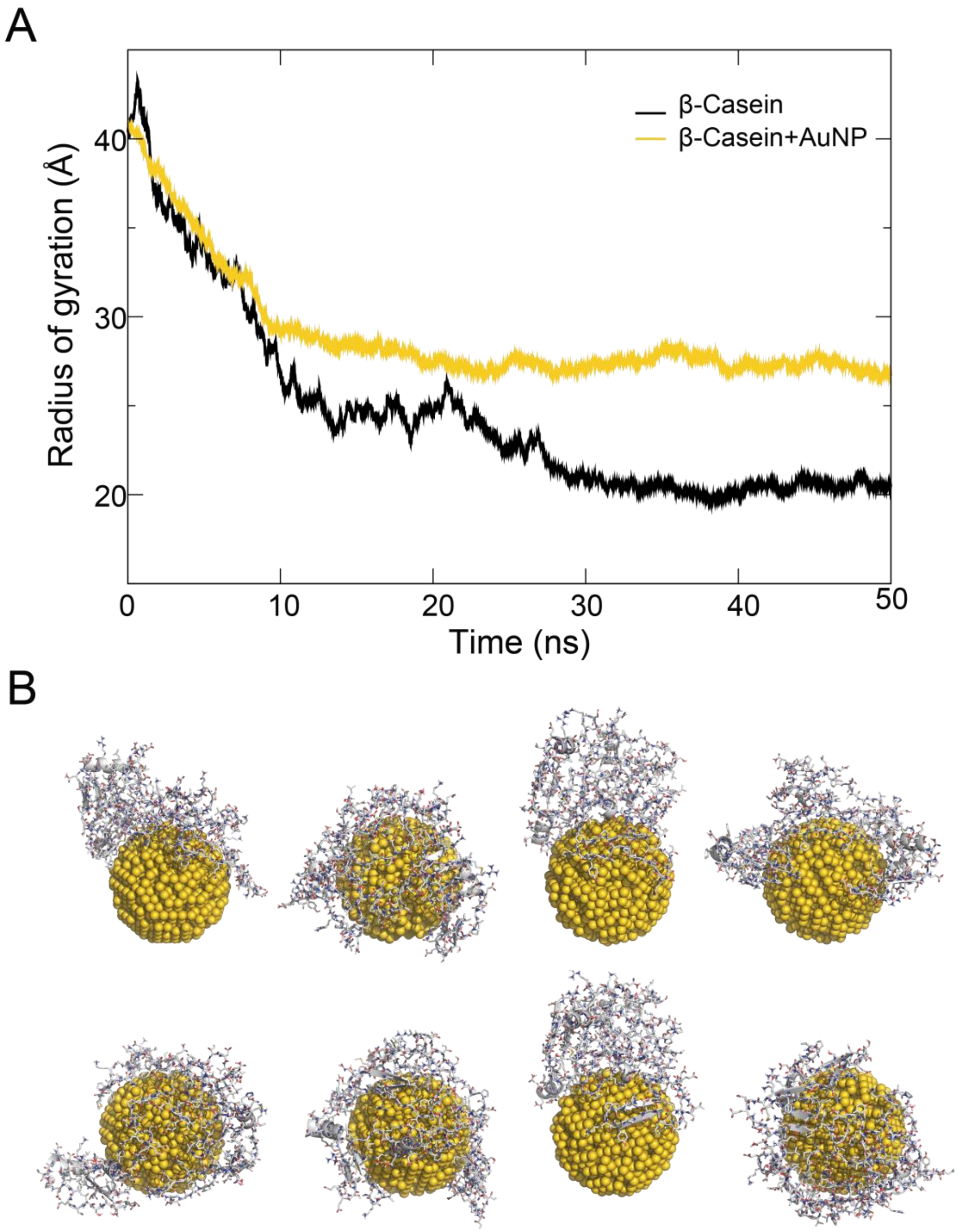


**Supplementary Figure 3. DMD simulations of βCas and AuNP.** (A) Radius of gyration (Rg) of βCas in the absence/presence of AuNP as a function of time. The thermo-fluctuations after 30 ns indicate the equilibrium of the simulation system and the larger Rg of βCas in the presence of AuNP indicates extended conformation of βCas. (B) Centroid structures of top 8 clusters of βCas and AuNP complexes, obtained from multiple independent DMD simulations with clustering analysis.

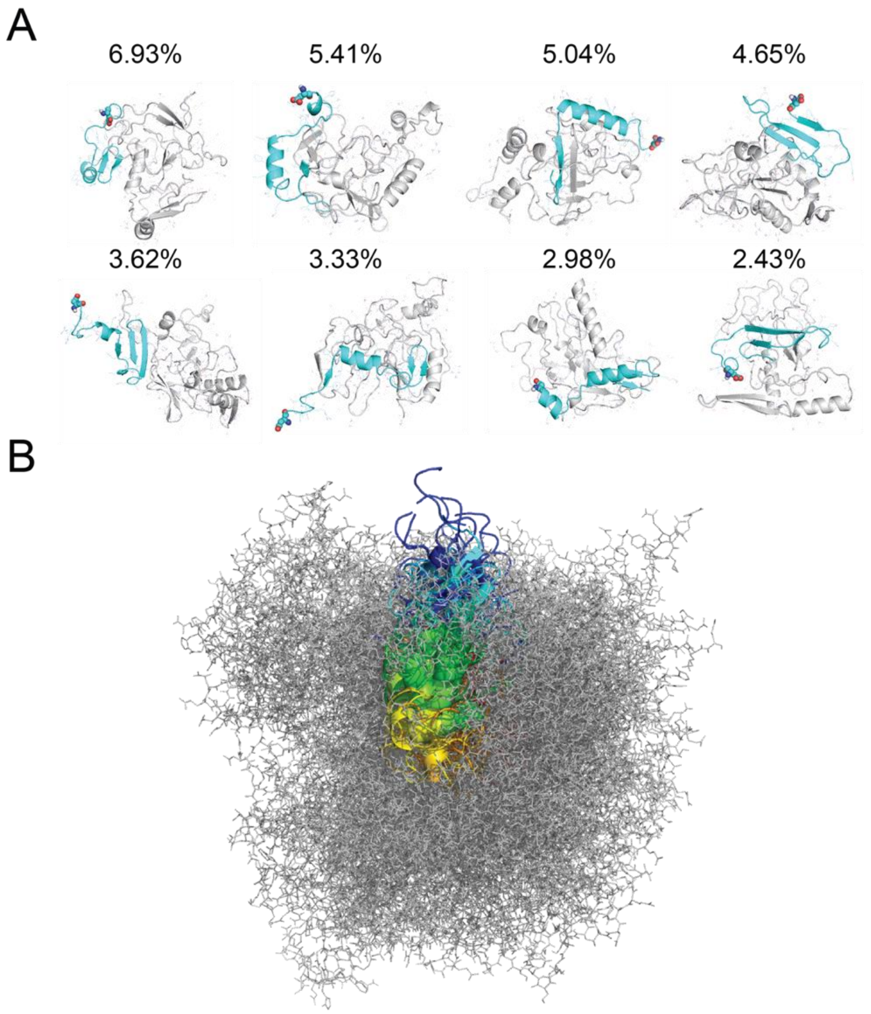


**Supplementary Figure 4. DMD simulation of βCas and Aβ monomer.** (A) Centroid structures of top 8 clusters of βCas and Aβ complexes. (B) Representative binding structures of Aβ (cartoon in rainbow color) and βCas (sticks in gray) aligned according to Aβ. The complex structures were obtained from independent binding simulations of Aβ and βCas monomers, and the centroid structures of top 50 clusters were used in the alignment.

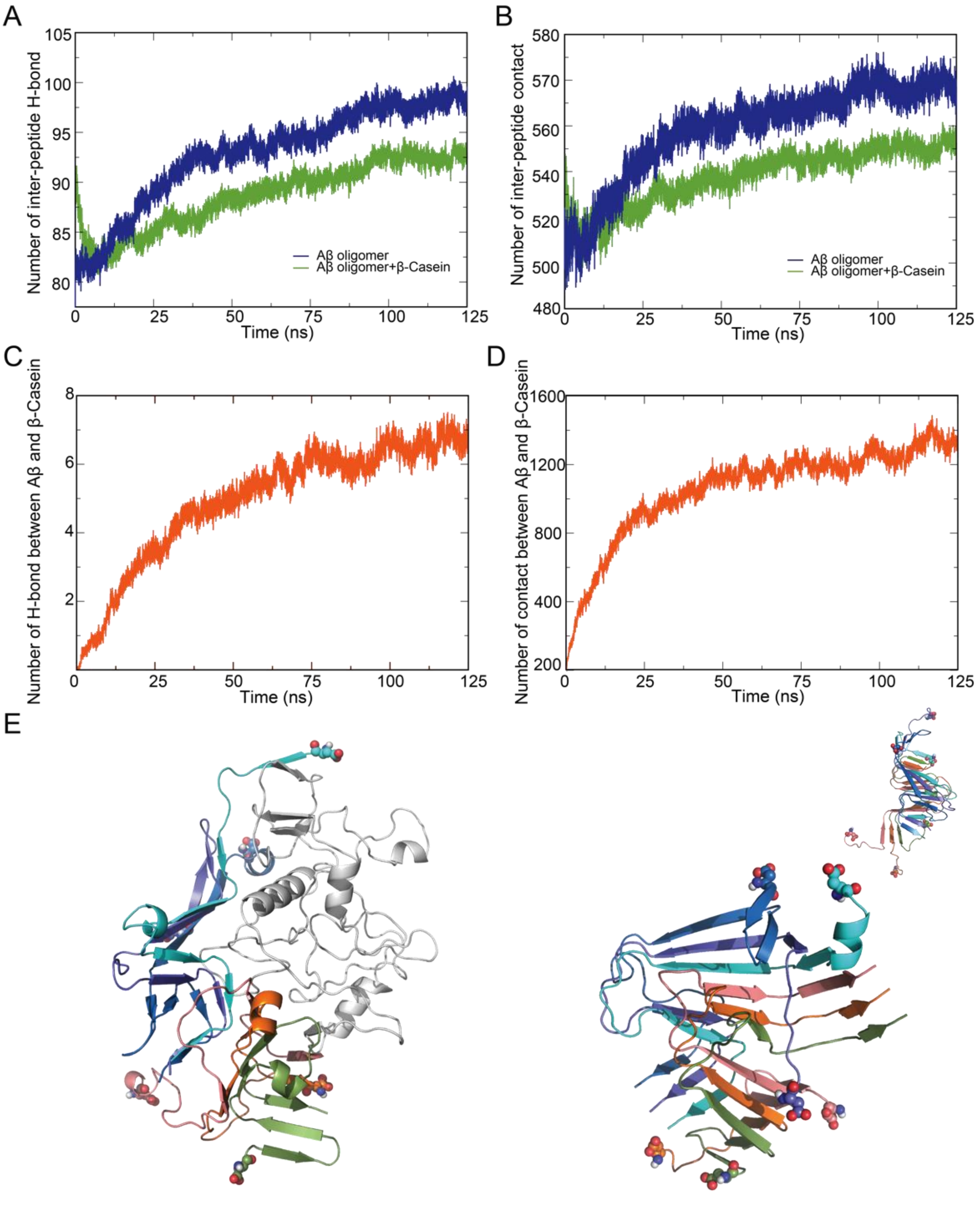


**Supplementary Figure 5. DMD simulation of βCas and Aβ oligomer.** Number of Aβ inter-peptide H-bonding (A) and contact (B) ranging from residues 10 to 42 in the presence/absence of βCas as a function of time. Number of H-bond (C) and contact (D) between Aβ oligomer and βCas as a function of time. (E) Representative snapshots of Aβ oligomer in the presence (left) and absence (right) of βCas (gray) after 125 ns of DMD simulations.

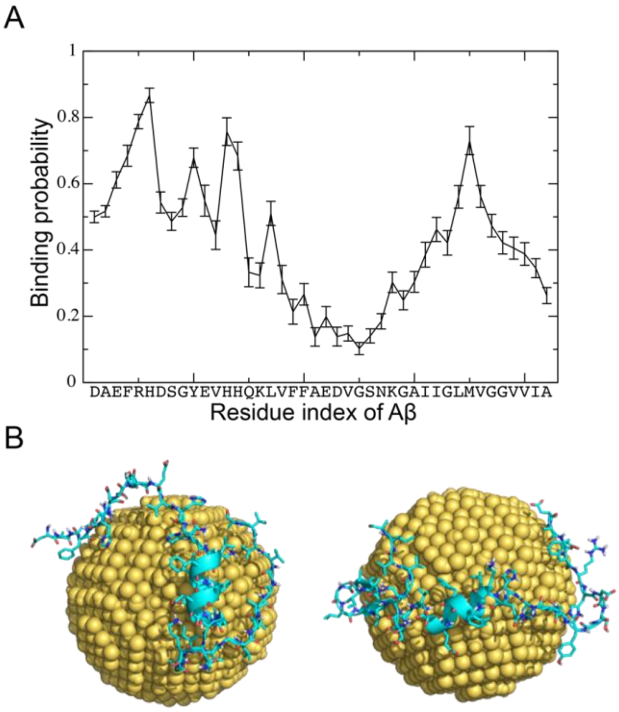


**Supplementary Figure 6. binding of Aβ with a bare AuNP.** (A) Binding probability of each Aβ residue with the AuNP. (B) Typical snapshots of Aβ binding with the AuNP.

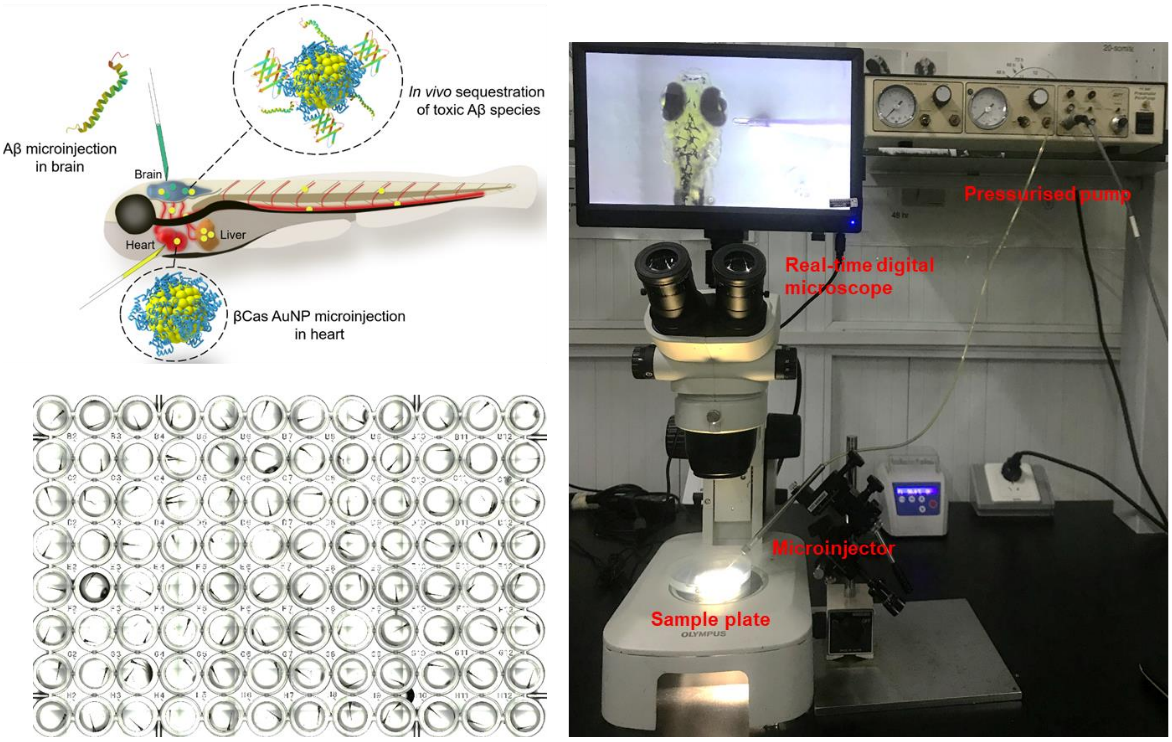


**Supplementary Figure 7.** High-throughput setup for Aβ microinjection with zebrafish larvae.

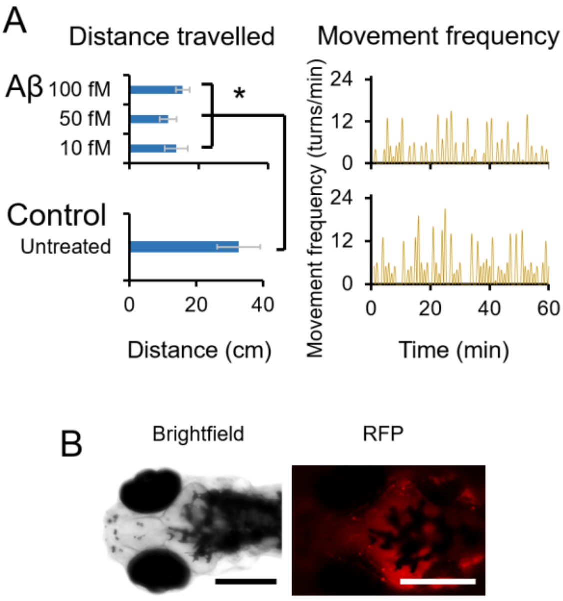


**Supplementary Figure 8. Behavioral changes in zebrafish larvae on the 3rd day post Aβ treatment.** (A) Zebrafish larvae swimming behavior was supressed significantly (*p<0.05*) on the 3rd day post Aβ treatment. Swimming distance was reduced to 13.5 ± 3.3, 11.1 ± 2.4 and 15.4 ± 1.9 cm with 10, 50 and 100 fM Aβ as compared to 35.3 ± 4.3 cm of untreated control (n=3). (B) Aβ treated larvae were injected with Congo red dye on the 3rd day post Aβ treatment and slight retention of the dye was observed in the brain. Scale bars in the images are 300 µm. Error bars represent the standard deviation. Source data are provided as a Source Data file.

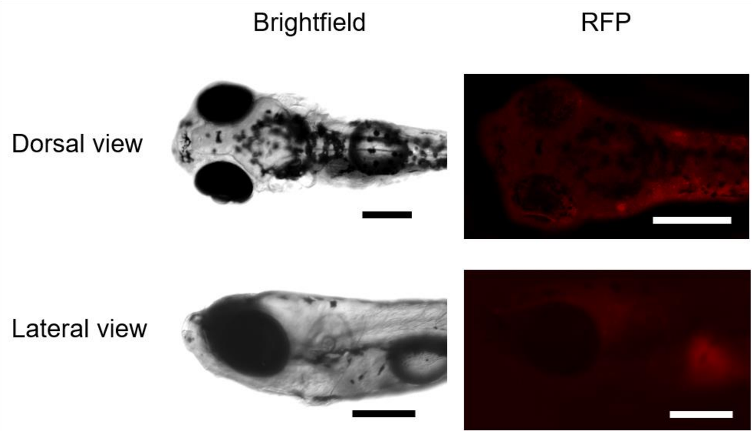


**Supplementary Figure 9.** Dorsal and lateral view of untreated zebrafish larvae under brightfield and RF channel of microscope. It indicates no background fluorescence in the larvae to interfere with the other fluorescence measurements. Scale bars in all images are 200 µm.

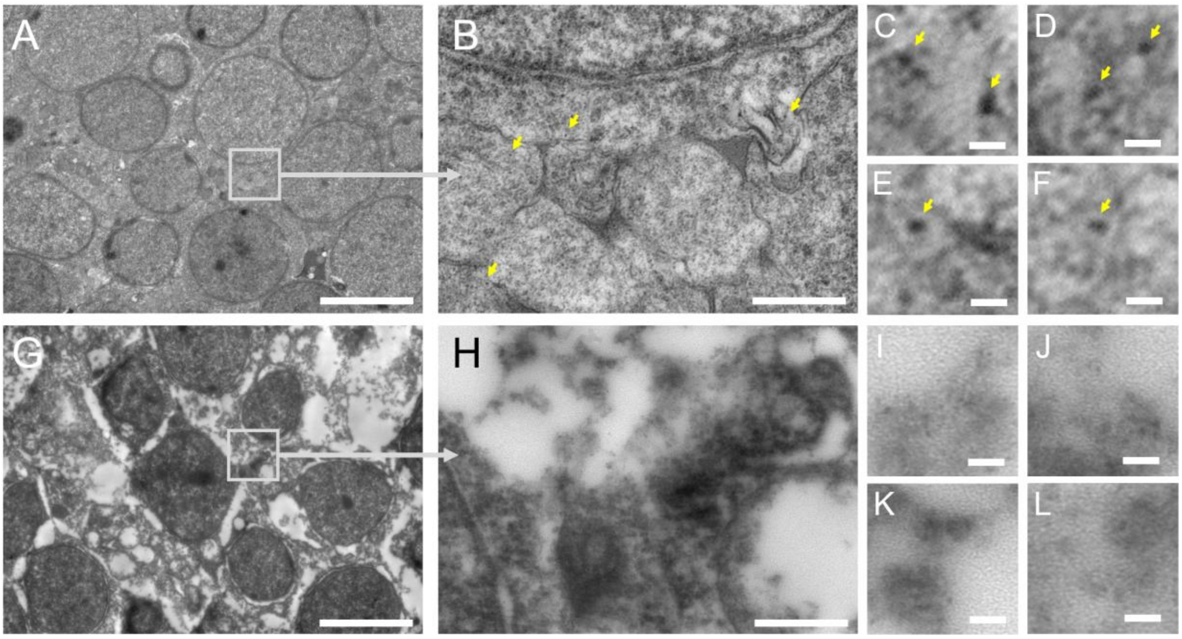


**Supplementary Figure 10. TEM imaging of the brain tissues of zebrafish larvae containing βCas AuNPs.** Zebrafish larvae treated with intracardiac injection of 3 ng Au equivalent βCas AuNPs were subjected to TEM analysis of the brain tissues for detection of AuNPs translocated across BBB. (A, B) TEM images of the brain tissues of βCas AuNPs treated larvae. Presence of AuNPs in the interstitial spaces are highlighted and presented as zoomed images (C-F). (G, H) However, untreated control larvae did not show any presence of AuNPs in the tissue sections (I-L). Scale bars in A,G: 5 µm; B,H: 500 nm and C-F,I-L: 10 nm.

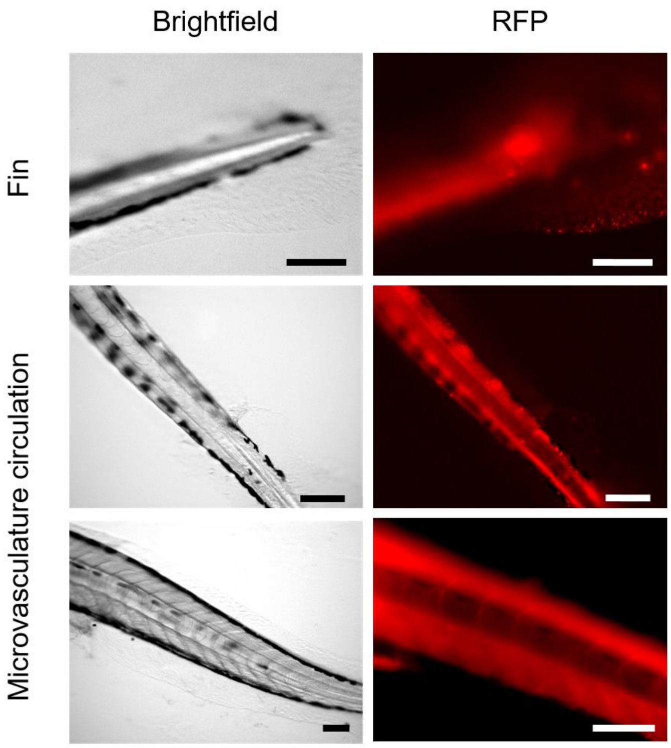


**Supplementary Figure 11. Biodistribution of βCas AuNPs.** βCas AuNPs were conjugated with neutral red and imaged for their biodistribution in zebrafish larvae. Red fluorescence of βCas AuNPs was traced in microvasculature circulation and fins of the larvae. Scale bars in the images are 200 µm.

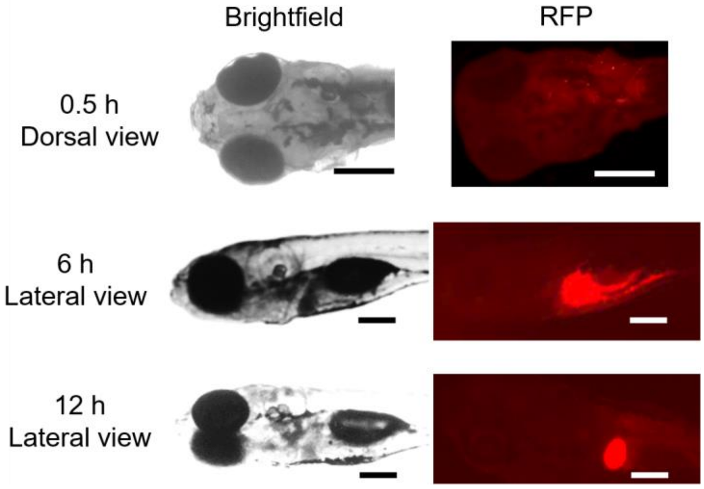


**Supplementary Figure 12. Biodistribution of βCas micelles in zebrafish larvae.** βCas micelles were conjugated with neutral red and imaged for their biodistribution in zebrafish larvae. No fluorescence was observed from the larval cerebral region in 0.5 h or later, indicating inability of large βCas micelles (~100 nm) to translocate across the BBB. Red fluorescence was observed from the hepatic region of the larvae at 6 and 12 h, indicating slow elimination of βCas micelles directly from the liver. Scale bars in images are 200 µm.

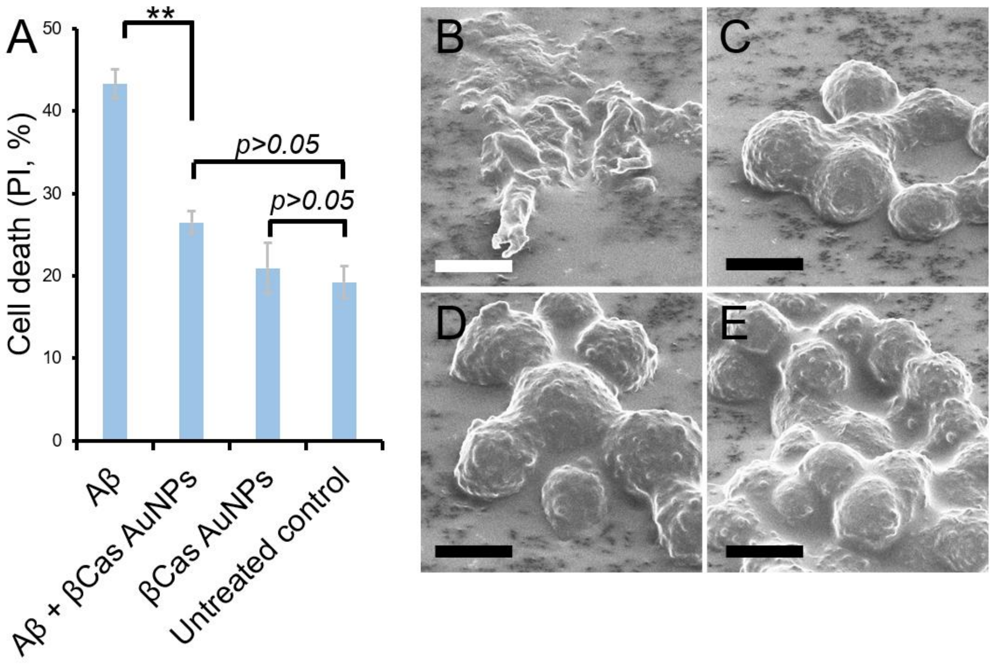


**Supplementary Figure 13. Mitigation of Aβ-induced SH-SY5Y cytotoxicity with βCas AuNPs.** (A) Cell viability assay (n=3) and helium ion microscopy (HIM) images of (B) Aβ treated (20 µM), (C) Aβ (20 µM) with βCas AuNPs (50 µM), (D) βCas AuNPs alone (50 µM) and (E) untreated control cells. Scale bars in TEM images are 4 µm. Error bars represent the standard deviation. Source data are provided as a Source Data file.

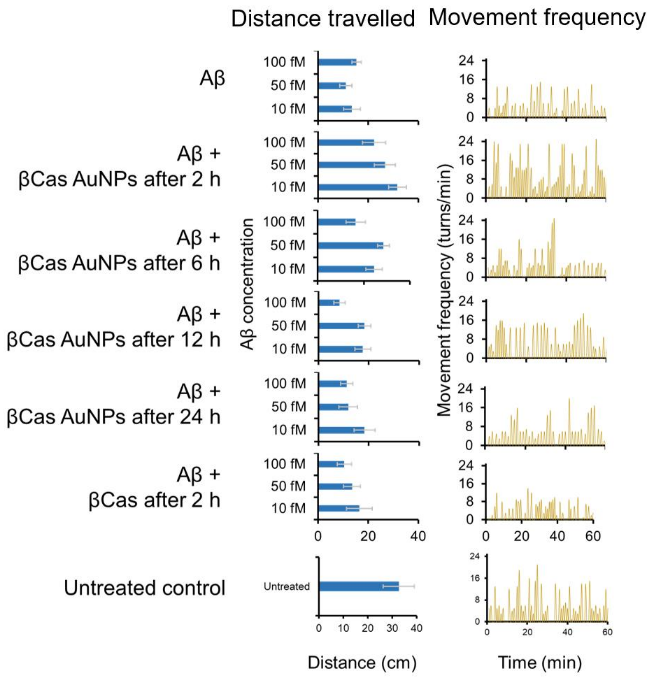


**Supplementary Figure 14.** Mitigation of Aβ induced behavioral abnormalities in zebrafish larvae by βCas AuNPs, on the 3rd day post Aβ treatment (n=10). Error bars represent the standard deviation. Source data are provided as a Source Data file.

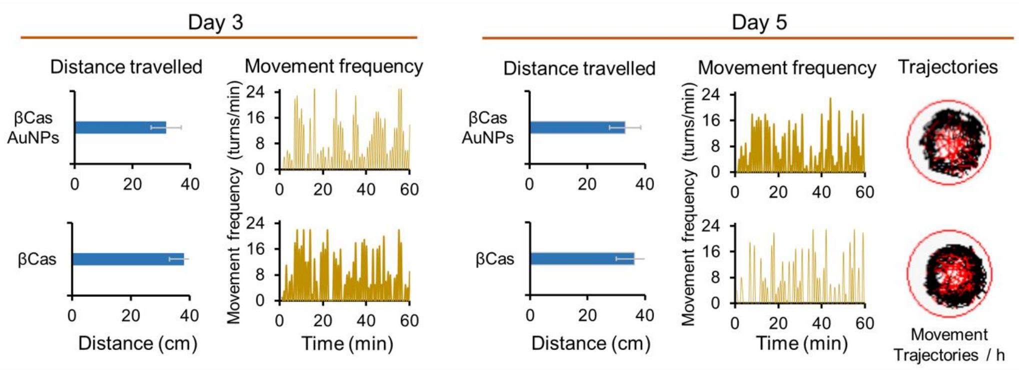


**Supplementary Figure 15.** βCas AuNPs and βCas did not influence the behavior of zebrafish larvae (n=10). Error bars represent the standard deviation. Source data are provided as a Source Data file.

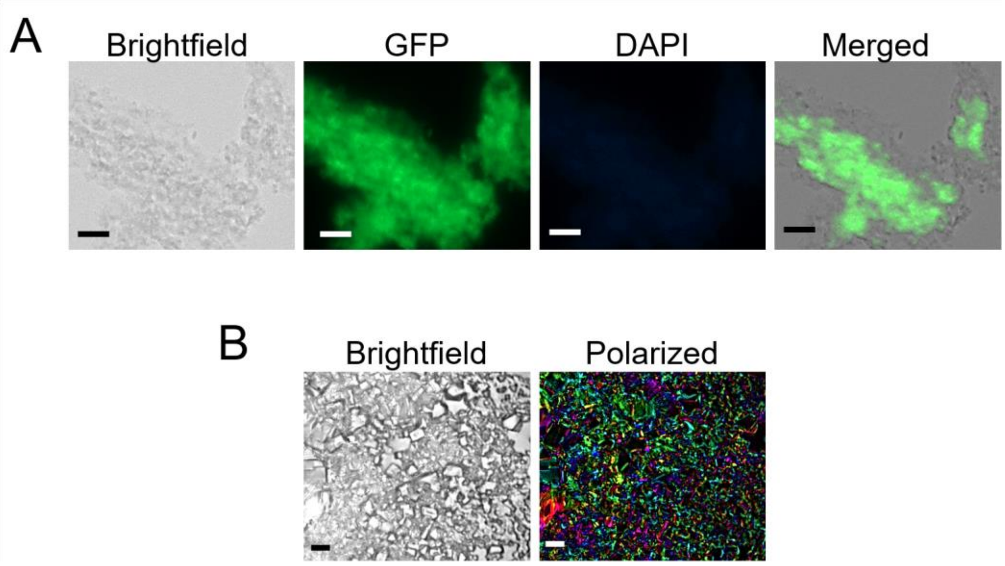


**Supplementary Figure 16.** Immunohistochemistry IHC (A) and polarized light microscopy (B) of fibrillized Aβ deposited on glass slides, performed as positive controls for IHC and polarized light microscopy. Scale bars in all images are 50 μm.

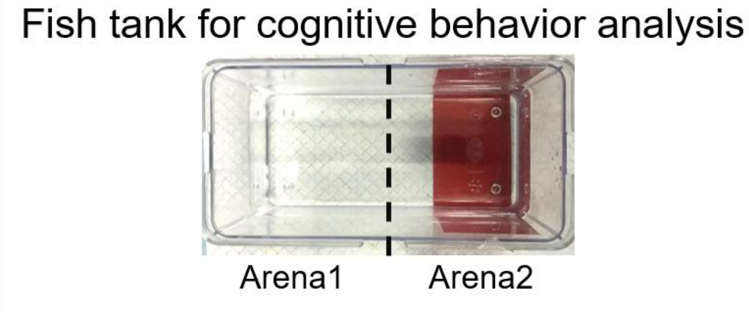


**Supplementary Figure 17.** Fish tank (1L) used for cognitive behavior analysis of adult zebrafish. The tanks were hypothetically divided into two arenas. Whenever, fish swims into arena 2, electrodes of 9V battery were dipped in the arena 2 to shock the fish and train it to remain in arena 1. Arena 2 was labelled with red color to associate with the punishment. 20 min training was performed, followed by 2 min of observation period when the electric source was removed. Observations were performed 3 times for each fish, 2 h apart (n=3).

## Supplementary References

1 Allen, M. P. & Tildesley, D. J. *Computer simulation of liquids*. (Oxford university press, 2017).
2 Rapaport, D. C. & Rapaport, D. C. R. *The art of molecular dynamics simulation*. (Cambridge university press, 2004).
3 Lazaridis, T. & Karplus, M. Effective energy functions for protein structure prediction. *Curr. Opin. Struc. Biol.* **10**, 139-145 (2000).
4 Ding, F., Borreguero, J. M., Buldyrey, S. V., Stanley, H. E. & Dokholyan, N. V. Mechanism for the α-helix to β-hairpin transition. *Proteins: Struct. Funct. Bioinf.* **53**, 220-228 (2003).
5 Andersen, H. C. Molecular dynamics simulations at constant pressure and/or temperature. *J. Chem. Phys.* **72**, 2384-2393 (1980).
6 Hughes, Z. E., Wright, L. B. & Walsh, T. R. Biomolecular adsorption at aqueous silver interfaces: first-principles calculations, polarizable force-field simulations, and comparisons with gold. *Langmuir* **29**, 13217-13229 (2013).
7 Manning, G. S. Limiting laws and counterion condensation in polyelectrolyte solutions I. Colligative properties. *J. Chem. Phys.* **51**, 924-933 (1969).
